# Optimization and Fabrication of Multi-Level Microchannels for Long-Term Imaging of Bacterial Growth and Expansion

**DOI:** 10.3390/mi13040576

**Published:** 2022-04-07

**Authors:** Hsieh-Fu Tsai, Daniel W. Carlson, Anzhelika Koldaeva, Simone Pigolotti, Amy Q. Shen

**Affiliations:** 1 Micro/Bio/Nanofluidics Unit, Okinawa Institute of Science and Technology Graduate University, Onna-son, Okinawa 904-0495, Japan; daniel.carlson@oist.jp; 2 Department of Biomedical Engineering, Chang Gung University, Taoyuan 333, Taiwan; 3 Biological Complexity Unit, Okinawa Institute of Science and Technology Graduate University, Onna-son, Okinawa 904-0495, Japan; anzhelika.koldaeva1@oist.jp (A.K.); simone.pigolotti@oist.jp (S.P.)

**Keywords:** multi-level microfluidic device, live cell imaging, long-term microscopy imaging, focus drifting, immersion oil viscosity, bacterial population dynamics, single-cell studies, *E. coli*, mother machine, computational fluid dynamics

## Abstract

Bacteria are unicellular organisms whose length is usually around a few micrometers. Advances in microfabrication techniques have enabled the design and implementation of microdevices to confine and observe bacterial colony growth. Microstructures hosting the bacteria and microchannels for nutrient perfusion usually require separate microfabrication procedures due to different feature size requirements. This fact increases the complexity of device integration and assembly process. Furthermore, long-term imaging of bacterial dynamics over tens of hours requires stability in the microscope focusing mechanism to ensure less than one-micron drift in the focal axis. In this work, we design and fabricate an integrated multi-level, hydrodynamically-optimized microfluidic chip to study long-term *Escherichia coli* population dynamics in confined microchannels. Reliable long-term microscopy imaging and analysis has been limited by focus drifting and ghost effect, probably caused by the shear viscosity changes of aging microscopy immersion oil. By selecting a microscopy immersion oil with the most stable viscosity, we demonstrate successful captures of focally stable time-lapse bacterial images for ≥72 h. Our fabrication and imaging methodology should be applicable to other single-cell studies requiring long-term imaging.

## 1. Introduction

Even simple bacteria such as *Escherichia coli* (*E. coli*) give rise to non-trivial phenomena as their population grows. For example, it has been observed that individuals are characterized by measurable phenotypic differences that persist through generations [1]. These differences, in particular fluctuations of cell division times, affect the population as a whole [2,3,4]. Further, when bacteria grow in space, they can align with their neighbor and generate complex spatial patterns [5,6]. These dynamics can affect the genetic diversity of the population [7]. Some of these effects can be quantitatively studied by means of microfluidic devices. Indeed, advances in microfabrication have enabled creation of microdevices with confined geometry and controlled microenvironments to study microbial dynamics at single-cell resolution, see, e.g., [8,9,10,11,12,13,14,15]. These devices can also be used to mimic microstructures, such as those found in soil, using controlled flow and microenvironmental conditions [16]. More complex microenvironments can be mimicked in multi-level microdevices for studying interaction between bacteria and human physiology, e.g., bacterial interaction with intestinal villi epithelial cells, in a quantitative manner [17,18,19].

However, building microdevices for bacterial studies presents its own challenges. For one, entrapping micron-sized bacteria that are sensitive to external flows requires optimized hydrodynamically confined design with feasible microfabrication methods [15,20,21,22,23]. A careful hydrodynamical design is essential for nutrient perfusion, waste removal, and prevention of bacterial biofilm formation to avoid microchannel blockage [24]. In addition, a robust stable imaging system is necessary to achieve long-term single-cell studies [25].

Microchannels hosting cells serve as a model system to mimic and study cell growth and competition in broad topics, such as biofilm formation in the soil and stem cell proliferation in the intestinal duct [19,26,27,28]. In the original work involving microchannels to entrap and study the outgrowth of single bacterium with one open end, also known as the mother machine, single cell genetics was investigated but competitions among bacterial clones could not be examined due to the limitation of diffusion-based nutrient delivery with one open and one closed-end microchannel design [8]. Hashimoto et al. developed microchannels with two open ends where bacterial growth rate was found to increase in certain clonal populations compared to those of the ensemble colony [2]. However, the microchannel dimensions (1.5–3 μm in width) in these studies were limited by the microfabrication processes (resolution-limiting lithography and isotropic wet etching) and the flow cell was not optimized to prevent microbubble formation, leading to unstable flow field in which high shear gradient could cause filamentation of bacteria [29].

In this study, we devise a straightfoward microfabrication process to create multi-level microstructures by employing a maskless aligner, a spin coater, and a hotplate. Our hydrodynamically-optimized microchannel design facilitates bacteria entrapment and culturing for more than 80 h. With this device, we have successfully imaged bacteria without focus drift by using a stable low viscosity immersion oil for over 60 h.

## 2. Materials and Methods

### 2.1. Design and Fabrication of Multi-Level Microchannel in Silicon Mold

**Figure 1:**
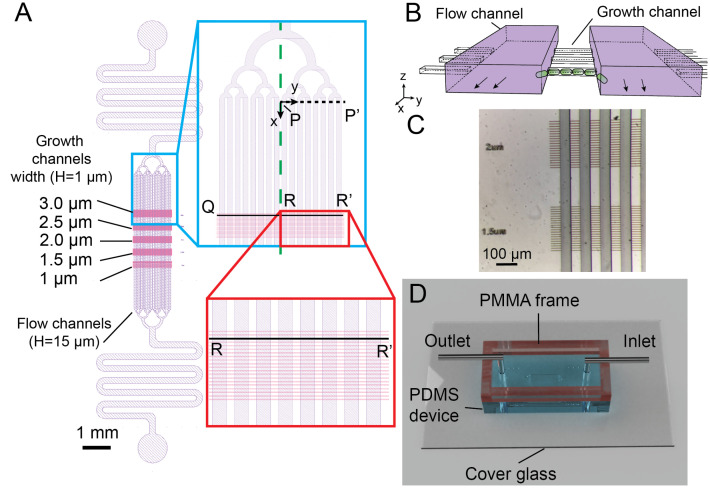
The microdevice containing multi-level microchannnels for long-term bacterial expansion imaging. (**A**) Schematic of the microchannel design in the PDMS device. Two layers are shown in purple (flow channels for nutrient delivery, 15 μm in height) and pink (growth channels for bacterial growth, 1 μm in height). The flow channels are joined by a tree-like flow stabilizing structure and a flow resistor to the inlet and outlet of the device. The green dashed line shows the symmetry axis used in OpenFOAM numerical simulation. The black dashed line (P-P’) indicates the region and the coordinate system used for numerical simulations in the flow channels. The zoomed-in part of the red box displays the growth channels (3 μm-wide) housing the bacteria. The black line segment (Q-R-R’) represents the growth channel analyzed in the numerical simulation (**B**). The perspective diagram of two flow channels (purple) and three growth channels illustrates their 3D relationship. The bacteria are cultured in the growth channels and old bacteria are expelled and sheared off in flow channels. The diagram is not to scale for sake of clarity. (**C**) Episcopic stereo-microscopy image of the silicon mold showing the high accuracy in aligning the two layers of the PDMS microchannels. (**D**) Snapshot of the assembled PMMA/PDMS microfluidic device for the bacterial experiments. The top reservoir encircled by the PMMA frame are filled with liquid buffer. Small air-bubbles are prevented from entering the chip and the hydrostatic pressure differences are mitigated to prevent secondary flow disturbing the bacteria.

The multi-level microchannel was designed in AutoCAD (AutoDesk, San Rafael, CA, USA) and exported to GDS format for maskless lithography using KLayout. The design consists of three layers: the first alignment mask layer, the flow channel layer, and the growth channel layer (Figure 1). The flow channel layer (for nutrient delivery and bacterial removal) was 15 μm in depth and the growth channel layer (for bacterial entrapment, outgrowth, and long-term imaging) was 1 μm in depth.

To create the microstructure mold, we developed a lithography approach using a single negative photoresist (mr-DWL-5, Micro Resist Technology GmbH, Berlin, Germany) and a maskless writer (DL-1000, Nanosystem Solutions, Okinawa, Japan). The hard baking temperature and exposure dosage protocol was based on the manufacture instructions. Briefly, after lithographic exposure, the wafer was hard baked and developed in propylene glycol methyl ether acetate (PGMEA, Sigma-Aldrich, Burlington, MA, USA), washed thoroughly with isopropanol, and dried with nitrogen air. The true depth of the microstructures was confirmed with a stylus profilometer (DektakXT, Bruker, Billerica, MA, USA).

### 2.2. Fabrication and Assembly of the PDMS/PMMA Microdevice

The silicone mold containing the multi-level microstructures was passivated with Trichloro(1H, 1H, 2H, 2H-perfluorooctyl)silane (Sigma-Aldrich, Burlington, MA, USA) in a vacuum dessicator for 2 h to render the surface hydrophobic and ease removal of mold. The passivated mold was placed in a custom polytetrafluoroethylene holder made in-house to cast 4 mm-high poly(dimethylsiloxane) precursor (PDMS, Sylgard 184, Dow Corning, Midland, MI, USA), a biocompatible transparent silicone rubber, containing the monomer:curing agent = 10:1 ratio mixed with an orbital mixer (ARE-310, Thinky, Tokyo, Japan). Then, 50 g of mixed PDMS precursor was poured on the multi-level mold, degassed in vacuum, covered with a transparent acrylic sheet, and cured at 80 ${}^{\circ }$C for at least 4 h to crosslink PDMS into gel form. Individual PDMS microdevice was diced, inlet and outlet holes were punched on the device with a puncher (21 gauge, Accu-punch MP, Syneo, West Palm Beach, FL, USA).

Due to the small working distance (0.13 mm) of 100× microscopy objectives required for bacterial imaging, high-precision coverglass (no.1.5H, 60 × 24 × 0.175 mm, Paul Marienfeld GmbH, Lauda-Königshofen, Germany) was used. The coverglasses were mounted on a 3D printed washing stand made in-house and ultrasonically cleansed in a detergent bath (TFD4, Franklab, Montigny-le-Bretonneux, France) and ultrapure water (MilliQ, Millipore, Billerica, MA, USA) before drying at 80 ${}^{\circ }$C.

The PDMS microdevice treated by an ambient air plasma (PDC-001-HP, Harrick Plasma, Ithaca, NY, USA) was irreversibly bonded to a coverglass. The microdevice was tilted 5–10${}^{\circ }$ to the glass edge to mitigate the interference patterns caused by microstructure induced diffractions, ensuring the focus locking mechanism on the microscope (Perfect Focus System, Nikon, Tokyo, Japan). A 2 mm-thick polymethylmethacrylate (PMMA) sheet was cut with a CO${}_{2}$ laser cutter (VLS2.30, Universal Laser Systems, Scottsdale, AZ, USA) and bonded with the PDMS microdevice with a dual energy double sided tape (5302A, Nitto Denko Corporation, Osaka, Japan) [30], completing the assembly of the PDMS/PMMA microdevice. To avoid biofilm formation and clogging of the 1 micron-high growth channels, a pristine microdevice was used for each experiment.

### 2.3. Bacterial Cultivation and Maintenance

The *E. coli* strain MG1655 derived from K-12 wild-type strain was used in this study. MG1655 was cultivated and maintained in Luria–Bertani agar or LB broth (LB Lennox, Becton Dickinson, Franklin Lakes, NJ, USA). To assist tracking bacteria of different lineages with fluorescence microscopy, MG1655 strain were transformed with a low copy plasmid constitutively expressing a green fluorescence protein (GFP) or mCherry red fluorescence protein (pUA66 PrpsL-GFP Kan${}^{R}$ or pUA66 PrpsL-mCherry Kan${}^{R}$). The pUA66 plasmid harbors a cassette containing a kanamycin resistance gene and a promoter sequence for 30S ribosomal protein upstream of the fluorescent protein open reading frame (ORF). The pUA66 PrpsL-GFP was a gift from Dr. Pamela Silver from Harvard University (Addgene plasmid #165606; accessed date: 5 April 2022; http://n2t.net/addgene:105606; RRID: Addgene_105606) [31]. Details on pUA66 PrpsL-mCherry production can be found in our recent work [7].

The pUA66 plasmid was transformed in MG1655 using standard chemical transformation with 0.1 M calcium chloride and 42 ${}^{\circ }$C heat shock. The transformed bacterial colonies bearing the plasmid were selected with 50 μg mL${}^{-1}$ kanamycin (Nacalai Tesque, Kyoto, Japan) in the LB broth or agar. The bacteria strain was preserved under −80 ${}^{\circ }$C following overnight outgrowth in LB broth and addition of sterile glycerol to a final concentration of 15 wt%.

### 2.4. Numerical Simulation

Both finite element method (FEM) and finite volume method (FVM) are commonly used numerical methods in computational fluid dynamics (CFD), differing in their spatial discretization schemes. Both methods perform similarly for a given accuracy, but FVM may require less memory than FEM under certain implementations [32,33,34]. Mature commercial software, such as the FEM solver COMSOL Multiphysics^®^ are very user friendly and suitable for rapid design iteration of complex systems. Therefore, we leveraged COMSOL for initial design iteration of the overall device, and used the FVM solver OpenFOAM considering a reduced geometry with a refined mesh for a final high-fidelity analysis of the hydrodynamic performance [35].

We assumed that the aqueous LB broth and dilute *E. coli* suspension in LB broth used in our experiments were Newtonian fluids, corresponding to the continuity equation (Equation (1)) and Navier-Stokes equation (Equation (2)),

(1) ∇·u=0,

(2) $$\rho \left(\frac{\partial u}{\partial t}+u\cdot \nabla u\right)=-\nabla p+\mu \nabla^{2}u+f_{b},$$

where u, *p*, and $f_{b}$ represent the velocity vector, pressure, and body forces (per unit volume) that act on the Newtonian fluid, respectively. Due to the small length scales associated with the microfluidic device and the maximum mass flow rate *Q* (10 μL min${}^{-1}$) on the inlet in our study, Equation (2) can be simplified and solved as steady-state laminar flow without effects from the body force and temporal terms. The Reynolds number Re = ρUW/μ≈11 with U=Q/A, *A* is the cross section area of the microchannel. We further imposed zero pressure boundary condition at the outlet, and a no-slip boundary (u = 0) at the channel walls. For OpenFOAM we also introduced a symmetry condition at the midplane (see Figure 1A).

We first investigated the entire 3D model with COMSOL for rapid design iteration of the flow channels. We kept the full detail of the geometry but partitioned it with nested subdomains about the growth channels to handle meshing the length-scale disparity, culminating in a total element count of $8\times 10^{7}$ under the COMSOL “fine” mesh sizing. The finest element edge length to gap ratio was 1/40 for both the flow channels and the growth channels. Steady-state laminar flow through the device was solved with a COMSOL batch routine running on 128 processors. To probe the flow profile in the growth channels, we extracted an x−y slice of the volume at z=0.5 μm (mid-plane of the growth channels).

For detailed validation of flow profiles in the growth channels, we employed FVM-based OpenFOAM software for a reduced geometry mesh, achieving a cell edge length to gap ratio of 1/530 in the growth channels and 1/180 in the flow channels. The finer resolution in the growth channel provided a better description of the flow profile.

### 2.5. Experimental Setup and Live Cell Microscopy

To expel microbubbles, the microchannels in the PDMS/PMMA microdevice were passivated first with 99.5% ethanol (Nacalai Tesque, Kyoto, Japan) and then with ultra-pure water. The microchannel was then passivated with a solution composed of 2 mg mL${}^{-1}$ bovine serum albumin (BSA, Nacalai Tesque, Kyoto, Japan) and 0.5 mg mL${}^{-1}$ salmon sperm DNA (Thermo Fisher Scientific, Waltham, MA, USA) for 30 min before washed away by phosphate buffered saline (PBS). The top reservoir enclosed by the PMMA frame was filled with PBS as well to avoid entrapment of air-bubbles at the inlet or the outlet.

Prior to seeding bacteria in the microchannels, MG1655 *E. coli* bacteria outgrown overnight in LB broth were inoculated to a new tube of 4 mL LB broth with 0.1 mM kanamycin. The *E. coli* suspension was grown at 37 ${}^{\circ }$C and 200 rpm in an orbital shaker until the bacteria reached the log phase with OD${}_{600}=0.2$. Then, 1 mL of bacterial suspension was centrifuged at 3000× *g* for 10 min and resuspended in a 50 μL LB broth. Then, 10 µL of bacterial suspension was injected into the PDMS/PMMA device with a micropipet. The top reservoir was filled with PBS to prevent hydrostatic pressure difference in the system [30], allowing bacteria to stay in the growth channels without disturbance.

A PDMS/PMMA microdevice was affixed on a holder in a stage-top incubator (WKSM, Tokai Hit, Shizuoka, Japan) on an inverted motorized epi-fluorescence microscope equipped with focus locking mechanism (Ti-E, Nikon, Tokyo, Japan). A dummy PDMS/PMMA microdevice was placed on the holder as well and affixed with a K-type thermocouple (ANBE MST Co., Kanagawa, Japan). The stage-top incubator increased the temperature of the microdevice to 37 ${}^{\circ }$C within 10 min and maintained this temperature onward.

A set of tubing with stainless steel tubes (21 gauge, New England Small Tube, Litchfield, NH, USA) were attached to a 25 mL glass syringe (Trajan Scientific and Medical, Victoria, Austria) filled with M9 media supplemented with 1 μM rifapentine, 0.1 mM kanamycin, 2 mM glucose, and 1× MEM vitamins (Sigma-Aldrich, Burlington, MA, USA) and an empty 20 mL plastic syringe (Terumo, Tokyo, Japan) [7]. Rifapentine were added to inhibit biofilm formation without affecting the bacterial activity [36]. The stainless steel tube connected to the media filled syringe was inserted into the inlet and the tube connected to an empty syringe was inserted into the outlet. The M9 infusion at 1.6 μL min${}^{-1}$ using a multichannel syringe pump (neMESYS, Cetoni GmbH, Korbussen, Germany) started after the bacteria inhabited stably in the growth channel. The perfusion rate doubled every 2 h until it reached 10 μL min${}^{-1}$. The gradual ramping of flow rate was essential to prevent sudden pressure change that could expel the bacteria from the growth channels.

For long-term imaging of bacteria, an 100× oil immersion objective (Plan apo λ, Nikon, Tokyo, Japan) with 1.5× intermediate magnification was used to take time-lapse images after appropriate immersion oil was placed between the objective and the bottom of the PDMS/PMMA microdevice. The depth of field of 100× objective was only 0.39 μm so a focus locking system was used to mitigate thermal drift over long-term imaging (PFS, Nikon, Tokyo, Japan). The bacterial expansion in the growth channels were imaged with mercury lamp excitation (Intensilight, Nikon, Tokyo, Japan) together with GFP or mCherry filter cubes (Semrock, Rochester, NY, USA). The images were acquired using a scientific complementary oxide semiconductor camera (sCMOS, Prime95B, Teledyne Photometrics, Tucson, AZ, USA) at an interval of 3 min.

### 2.6. Viscosity and Refractive Index Measurements of Immersion Oils

We investigated the dynamic viscosity and refractive index of commonly used immersion oils, including Nikon NF2, Nikon F, Nikon F2, Olympus Immoil-F, and Millipore 104699 oil. The dynamic viscosity of the immersion oils were measured using a strain-controlled rotational rheometer (ARES-G2, TA Instruments, New Castle, DE, USA) with a stainless steel 50 mm cone-plate geometry (1${}^{\circ }$ angle). Immersion oils of 650 μL were dispensed using a positive displacement micropipette (Microman, Gilson, Middleton, WI, USA). The rheometer fixture was covered by a solvent trap and all the measurements were performed at 37 ± 0.1 ${}^{\circ }$C controlled via the Advanced Peltier System (TA Instruments, New Castle, DE, USA). The shear viscosity η was measured at steady state with the shear rate $\dot{\gamma }$ being varied from 0.01 to 100 s${}^{-1}$ with logarithmically increasing steps. To create aged oil samples, the pristine immersion oils were first dispensed into 1.5 mL microcentrifuge tubes with loose lids and later incubated for 60 hours on a 37 ${}^{\circ }$C dry bath. The aged oil samples were then loaded and measured on the ARES-G2 rheometer following the same protocol as the pristine ones.

The refractive index of immersion oils were measured by an automatic refractometer (Abbemat MW, Anton Paar GmbH, Graz, Austria) at 23 ${}^{\circ }$C and 589.3 nm (nD). The refractometer was calibrated by two-point adjustment with industrial reference standards (Kyoto Electronics Co., Ltd, Kyoto, Japan). Half a milliliter of pristine or aged immersion oil samples were dispensed into the sample well and the temperature was allowed to equilibrate before the results were taken. Two-way ANOVA with Tukey’s post-hoc test was conducted and null hypothesis was tested.

## 3. Results

### 3.1. Simplified Multi-Level Microstructure Fabrication by Maskless Lithography

The multi-level microchannel design consisted of a 15 μm-depth flow channel layer (purple, Figure 1A) for delivering nutrient and removing bacteria, overlaid on top of 1 μm-depth growth channels (pink, Figure 1A) with varied widths (1 μm to 3 μm) in which bacteria were retained and outgrown in a stable fashion (Figure 1B). The top flow channel layer contained 16 flow channels (Length L× Width W = 4500 × 50 μm) which branched uniformly into tree-like microstructures from inlet and outlet with flow resistors. The 30 μm-long growth channels with different widths (1 μm to 3 μm, 0.5 μm incremental depth, 10 channels per set with 10 μm inter-spacing) were designed so that bacteria could outgrow and expel from both ends, in contrast to the single-ended design of the mother machine [8,37].

The large aspect-ratio differences of the two channel layers (Figure 1B) often require microfabrications with two processes: one for the flow channel layer and the other for the growth channel layer [2,10,38,39,40]. We developed a simple lithography approach using a single negative photoresist and a maskless writer (DL-1000, Nanosystem Solutions, Okinawa, Japan). Although there were two layer structures, the small 1 μm thickness of the first layer created minimum contrast, making it difficult to be detected by the on-board camera in the maskless writer. To resolve this issue, we used an additional layer with cross marks for alignment. First, a 5 μm-thick photoresist was spin-coated on a 100 mm silicon wafer. Several 50 × 200 μm cross marks were placed at the center of the wafer and exposed at 405 nm by the maskless writer. To fabricate the 1 μm-high growth channel layer, the mr-DWL-5 photoresist was diluted with gamma butyrolactone (Sigma-Aldrich, Burlington, MA, USA) at 7.5:1 (*w*/*w*) ratio and spin-coated at 7500 rpm 30 s. The flow channel was aligned to the cross marks and exposed with 2× subpixel exposure followed by a post-exposure bake at 95 ${}^{\circ }$C for 5 min.

After the wafer cooled down to room temperature, without development of the flow channel layer, the mr-DWL-5 photoresist (15 μm) was spin-coated on the wafer at 750 rpm for 30 s and soft baked at 95 ${}^{\circ }$C for 5 min. The flow channel pattern was also aligned to the cross pattern, exposed with the maskless writer, and baked at 95 ${}^{\circ }$C for 5 min. The entire wafer was then developed in PGMEA and washed thoroughly with isopropanol and dried with nitrogen air. No visible defect was found in the microstructures made with the diluted photoresist. By aligning to the cross marks, good alignment could be achieved despite repeated photoresist coating (Figure 1C). The alignment process and exposure process were carried out directly in the maskless writer, therefore simplifying the workflow and the mold was created within one working day. Finally, PDMS microdevices were fabricated by standard molding process from the aforementioned mold and bonded to a coverglass with acrylic frame attached to the PDMS (details in the Section 2.2 in Materials and Methods).

### 3.2. Optimal Loading and Stable Culture of Bacteria by Balancing Hydrostatic Pressure and Optimizing Flow Resistance in the Microdevice

Due to the small height of the growth channels, it is difficult to introduce a single layer of bacteria inside the growth channels during loading. By balancing the hydrostatic pressure through filling liquid buffer in the top reservoir encircled by the PMMA frame (Figure 1D) [30], hydrostatic pressure-driven secondary flow could be prevented so that bacteria would swim into the growth channels in a stable manner.

In addition, pressure differences across two flow channels could cause pressure-driven flows to remove bacteria from the growth channels [2,10]. To address this challenge, the flow resistors and tree-like microstructures were introduced in our design as they were essential for uniform and stable flow distribution to keep the bacteria from escaping in the open-ended growth channels (Figure 1A,B).

**Figure 2:**
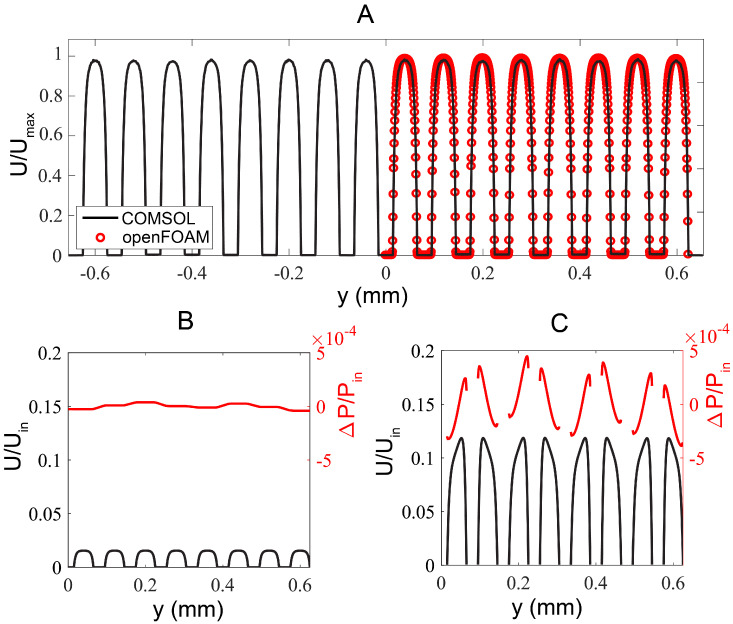
Numerical simulation results. (**A**) Flow profile comparison obtained from the full domain COMSOL solution (corresponding to Q-R’ segment in Figure 1A) and the reduced geometry OpenFOAM case, corresponding to R-R’ at x=452 μm, z=7.5 μm. (**B**) Flow and pressure profiles extracted from mid-plane of a growth channel (along R-R’ in Figure 1A; x=452 μm, z=0.5 μm). Secondary flow effects are negligible resulting in a nearly flat pressure distribution along the growth channels. The low pressure difference ensures that the bacteria are retained in the growth channels while strong shear takes place at the junction of growth channels and flow channels. (**C**) Flow and pressure profiles extracted at the *z* mid-plane immediately after the expansion section (along P-P’ in Figure 1A; x=0 μm, z=7.5 μm).

We further optimized and validated the microchannel design by performing computational fluid dynamics analysis. First, we investigated the full domain of the microfluidic design and optimized the flow uniformity across all 16 flow channels by the flow resistors and tree-like microstructures using FEM-based COMSOL simulation (Figure 1A). Flow velocity differences across different flow channels were less than 0.8% (Figure 2A). However, due to the large length-scale differences between the flow channel and the growth channel, mesh joining, and quality assurance became challenging, resulting in a coarse spatial resolution around the growth channels.

To better resolve flow in the growth channels, we next numerically investigated a reduced geometry using FVM-based OpenFOAM by assuming symmetry across the mid-plane of *x*–*y* plane (green dashed line, Figure 1A). We removed the outlet contraction such that only the inlet expansion and eight flow channels of length x=810 μm remained in the simulation. We considered only four rows of the 3 μm-wide growth channels starting at x=452 μm. The reduced geometry was then taken as the input to the snappyHexMesh utility, whereby an initial uniform background mesh was “snapped” to the extents of the geometry and incrementally refined to form the desired hexahedral mesh. A no-slip boundary condition was imposed on all channel walls, and uniform velocity and zero pressure conditions were applied to the inlet and outlets, respectively. Three iteratively finer meshes were generated this way, up to a final cell count of $1.33\times 10^{8}$ (mesh M3). Compared to the global COMSOL solution, the velocity profile noise floor about the growth channels was 3.5 times lower for M3. The velocity profile in the flow channels resolved by OpenFOAM (red symbols) coincided nicely with those by COMSOL (black curve), see Figure 2A.

Investigating the flow at the mid plane of a growth channel (R-R’ at z=0.5 μm in Figure 1A), the flow profile in individual flow channel was uniform and laminar (black curve in Figure 2B). Furthermore, secondary flow effects across each flow channel segments from two flow channels were negligible as evidenced by the nearly flat pressure distribution along the R-R’ profile (red curve in Figure 2B). The low pressure difference ensured that bacteria were retained in the growth channels while the high shear rate at the junction of the flow channel and growth channel enabled easy removal of the bacteria expelled from the two open-ends of the growth channels.

Figure 2C displayed the flow and pressure profiles in eight symmetrical flow channels at the mid-plane immediately after the inlet tree-like expansion (x=0μm, z=7.5μm). The uniform flow velocity and comparable pressure between individual channels suggested that the tree-like microstructures helped to distribute the flow uniformly among the flow channels.

**Figure 3:**
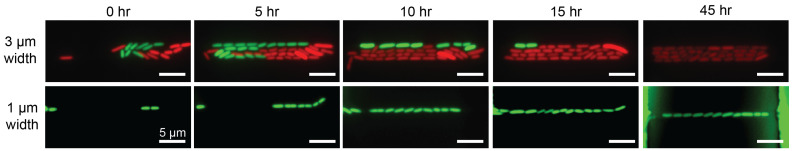
Stable bacterial expansion in 3 μm and 1 μm wide growth microchannels. Images are captured from 0 to 45 h in this specific study. (**Top panel**) Two bacterial populations individually carrying green or red fluorescence proteins compete in 3 μm-wide channels. After 15 h, the descendants of red fluorescence clone occupy the entire microchannel. (**Lower panel**) Outgrowth of *E. coli* carrying green fluorescence in a 1 μm-wide channels throughout 45 h. See also Ref. [7].

By hydrodynamic stabilization through the flow resistors and the tree-like microstructures in the microfluidic channels (Figure 1A) and the bubble-preventing world-to-chip interface design (Figure 1D), we have successfully developed a robust microfluidic device for bacteria culture and growth studies. The bacteria could be retained in the growth microchannels of 3 μm in width (top panel in Figure 3) or 1 μm in width (bottom panel in Figure 3) from the initial seeding and expanded to form clonal populations after perfusion for 60 h.

### 3.3. Choice of Immersion Oil for Long-Term Imaging of Bacterial Expansion in the Microdevice

Although the optimized microfluidic device we developed had enabled the bacteria to be cultured and grow consistently for more than three days (see Supplementary Video S1–S3), the microscopy focus often drifted after 10 hours during the long-term imaging session when the recommended immersion oil (Nikon F type) was used for the Nikon Ti-E microscope system.

The focus locking mechanism is essential for long-term microscopy imaging of biological specimen. Commercial microscope system equipped with focus locking mechanism utilizes an infrared light projection and detect its reflection at a glass–air or glass–liquid interface [41]. Any drift caused by thermal variation in the optical system or mechanical flexing of the sample can be corrected by refocusing along z-direction relatively to the interface with the motorized focusing stage. The 100× oil immersion objective (numerical aperture NA = 1.45) used in this study only has a depth of field of 0.39 μm and the *E. coli* bacteria are around 1 μm in size, therefore, if the focus locking mechanism is suboptimal or the glass–liquid interface contrast is not strong enough for detection, e.g., interfered by the refractive index difference between glass (1.516) and PDMS (1.43) [42], the image can easily drift out of focus or oscillate around the focal plane (ghost effect). The poor focus in images is the major cause of image segmentation failure in single cell tracking [43].

**Figure 4:**
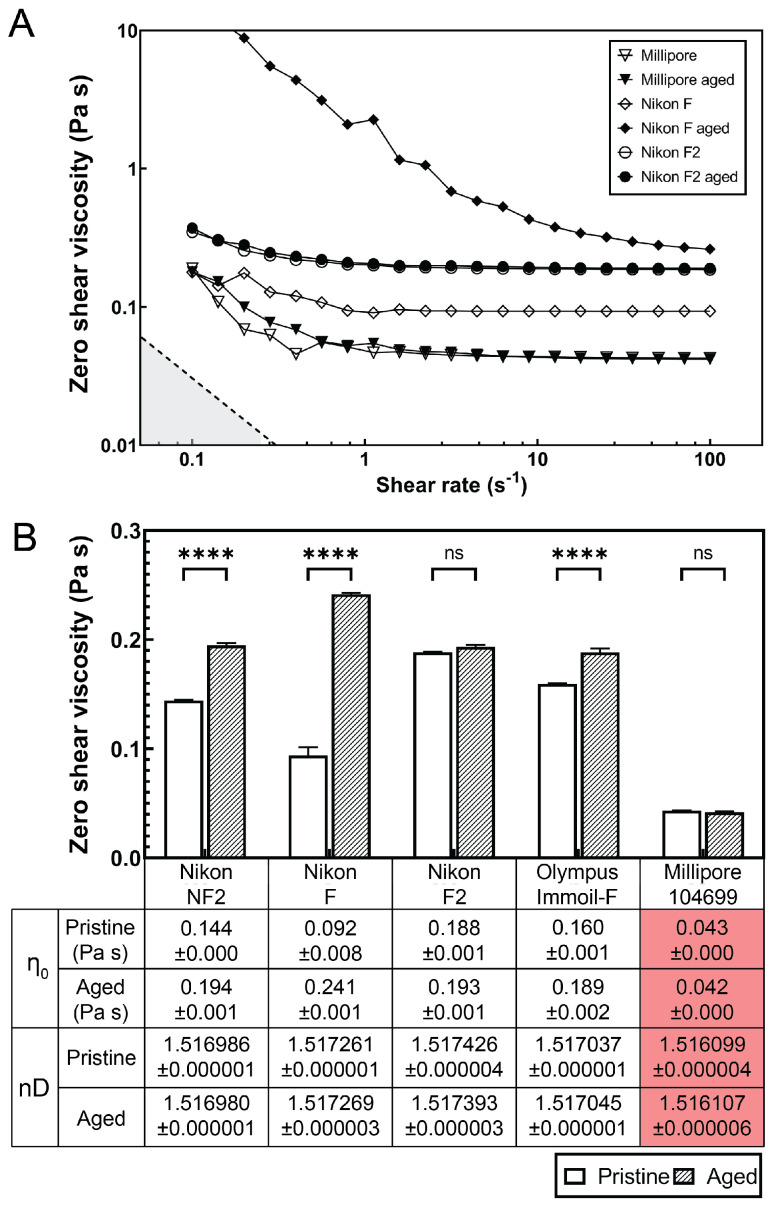
Dynamic viscosity and refractive index of microscopy immersion oils. (**A**) Shear viscosity-shear rate plot of pristine microscopy immersion oil (open symbols) and aged (60 h, closed symbols) at 37 ${}^{\circ }$C measured by a rotational shear rheometer. Nikon F oil (diamond symbols) shows a significant viscosity increase after aging while Nikon F2 and Millipore oil display minimum viscosity difference between pristine and aged samples. (**B**) Zero shear viscosity approximated by best-fit flow calculated from three measurements. The mean zero shear viscosities are listed in the table. The error bars indicate standard deviation. **** denotes p<0.0001; ns (non significant) denotes p>0.05. The refractive indexes nD (measured at 589.3 nm, 23 ${}^{\circ }$C) of pristine or 60 h-aged microscopy immersion oils are listed in the table.

To understand the role of the immersion oil in the focus locking function of the microscopy imaging system, we investigated the dynamic viscosity and refractive index of commonly used immersion oils, including Nikon NF2, Nikon F, Nikon F2, Olympus Immoil-F, and Millipore 104699 oil. The dynamic viscosity of the immersion oils were measured using a strain-controlled rotational rheometer (ARES-G2, TA Instruments, New Castle, DE, USA) with a stainless steel 50 mm cone-plate geometry (1${}^{\circ }$ angle). Figure 4A only displayed the flow curves of three representative immersion oils in their pristine and aged states. The dashed line in Figure 4A represents the limit of measurable viscosity defined by the instrumental torque limit [44,45],

(3) $$\eta >\frac{F_{\tau }T_{min}}{\dot{\gamma }},$$

where for cone-plate geometry, $F_{\tau }=3/\left(2\pi R^{3}\right)$, R=25 mm, and the minimal measurable torque on ARES-G2 rheometer $T_{min}=0.1\;\mu$N m. All the measured viscosity data were higher than the detectable viscosity at respective shear rates, suggesting that measurements were not affected by the inertia of the rheometer.

We found that the viscosity of the Nikon F type immersion oil increased by 262.5% after 60 h of aging under 37 ${}^{\circ }$C (similar imaging condition in long-term bacteria expansion studies), see Figure 4B. Other immersion oils, such as Olympus Immoil-F and Nikon NF2, exhibited 18.2% and 34.5% of viscosity increases after aging. However, the Nikon F2 type immersion oil and Millipore 104699 immersion oil did not show significant viscosity changes after aging (Figure 4B).

Further characterization of the optical properties (refractive index) of all the immersion oils before and after 60 h of aging showed little difference, suggesting that the focus drift was mainly caused by the viscosity increase in the immersion oil over the long-term imaging period (Figure 4B).

**Figure 5:**
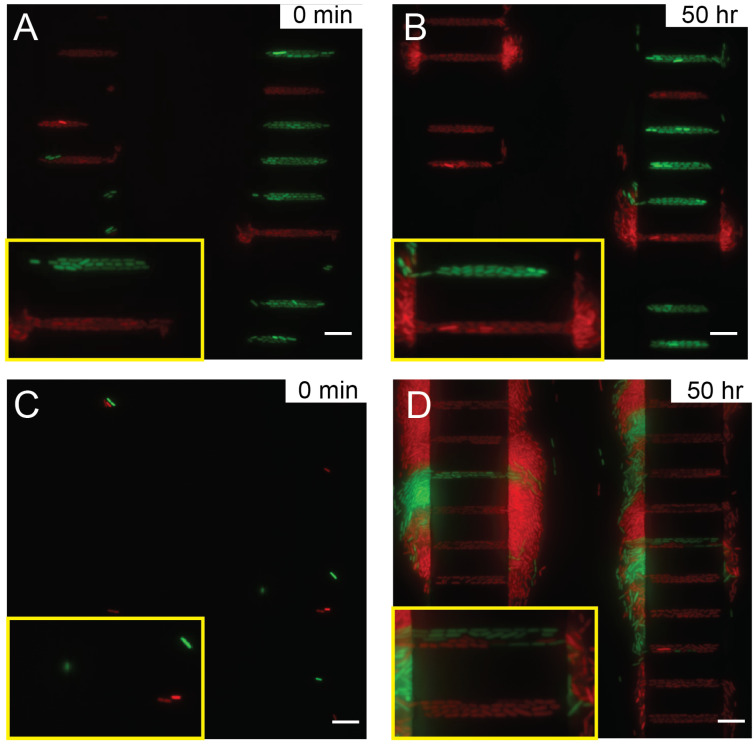
Imaging single bacteria expansion with Nikon type F immersion oil at (**A**) 0 min and (**B**) 50 h, and with Millipore immersion oil (**C**) at 0 h and (**D**) 50 h. The scale bars correspond to 10 μm. The yellow insets are enlarged views of two growth channels to highlight the focus quality.

We hypothesize that during the long-term imaging experiment, certain immersion oils are prone to aging, consequently their viscosities increase over time. Although the microscopy imaging system attempted to correct the focal length, the immersion oil with increased viscosity dragged the sample during Z stage movement, leading to error. As a result, the microscope focus gradually drifted over time even with focus locking mechanisms, see Figure 5A,B with Nikon type F immersion oil. Poorly focused images eventually contributed to inaccuracy or failure of data analysis. When using an immersion oil with low viscosity and no aging-associated changes, such as Millipore 104699 immersion oil, the focus drift was eliminated, see Figure 5C,D with Millipore immersion oil. Clearly focused images of bacterial expansion were acquired over more than three days (Supplementary Video S3). Note that individual bacteria with clear boundary could be seen clearly, even though the intensity was a bit lower than those in Figure 5B. Use of microscopy immersion oils whose viscosity does not change much with aging, such as Millipore immersion oil, has provided good focus stability as required for long-term imaging.

## 4. Discussion

We have designed and validated numerically that the open-ended microchannels with various widths are suitable for cultivating and tracking bacterial expansion in long-term studies in a high throughput manner. In particular, previous studies were limited to single-width microchannels due to microfabrication limitations. These microdevices were only suitable to study growth expansion of one bacterium [8,40] or clonal expansion of three lanes of bacteria [2], limiting the experimental throughput.

Note that the microdevice in this study is designed for bacterial expansion and not for trapping of single bacterium using nanostructures [46,47] or microdroplets [48]. The open-ended microchannels in our design confine the degree of freedom of bacterial expansion while allowing bacteria to interact with each other. The bacteria grow and align horizontally into organized stripe patterns along the wall and eventually get expelled into the flow channel (Figure 3). A study of the clonal expansion and its mathematical model can be found in Ref. [7].

Although *E. coli* bacteria are randomly loaded into the growth channels in our microdevice by their active swimming and passive diffusion, single or multiple bacteria can form the colony in each growth channel. How the growth dynamics and cell–cell interactions in confined geometry may be affected by multiple bacterial strains with different phenotypes will be an interesting topic worth future investigations.

Drying of the immersion oil has been an issue during microscopy imaging, as dried oil must be prevented from depositing on the microscope lens [49,50]. In addition to the drying effect, our studies also reveal that certain immersion oils become more viscous over time, leading to focus drift and ghost effect during long-term imaging. Our results suggest that the choice of low-viscosity immersion oil without aging-associated viscosity change is vital for long-term bacterial imaging which requires exceptional focus locking performance. Future microbial or single cell imaging studies that require long-term observations with oil immersion lens can benefit from these findings.

Combining hydrodynamically-optimized microchannels and the use of optimal immersion oil, we have developed a high throughput platform to study bacterial growth and expansion in confined geometry. Bacteria without genetic manipulation other than fluorescence expression can be stably cultured in the microchannels. For future work, addition of active control components in the microdevice, such as microvalves, can provide active manipulation or trapping of bacteria [40]. Due to the use of wild type bacteria, long-term culture in microsystems inevitably leads to biofilm formation (Figure 5) despite channel passivation and use of low dosage antibiotic rifapentine. In the future, inhibition of biofilm formation by new antibiotics and new anti-fouling treatment might further increase the imaging duration of our platform.

Furthermore, our design strategies and device fabrication techniques can be applied to general studies of single-cell growth and cell–cell interactions in confined geometry [51]. For example, confined channels can mimic the porous microstructures in the soil to study how bacteria grow, adapt, and evolve in settings otherwise difficult to observe in nature [6,16,28,52,53]. In addition, the mechanical interaction due to single cell growth is not limited to prokaryotes, such as bacteria. In eukaryotic tissues, such as the epiderm or the intestinal duct, stem cells reside in specific stem cell layers, outgrow, and differentiate as the tissue develops [26,54]. Designing and optimizing microfluidic platforms to model these systems can shed new insights into their cellular dynamics.

## 5. Conclusions

In this study, we developed a process to align and fabricate multi-level and multi-width microchannels for long-term bacterial expansion studies. We optimized the microchannel design by balancing hydrostatic pressure and flow resistance in the microdevice, supported and validated by CFD analysis. As a result, we have achieved a stable culture of bacteria in open-ended microchannels. The hydrodynamical optimization of our microdevice can be adapted for designing new devices to study bacterial dynamics in microenvironments and a variety of other single-cell studies. Our findings on material property changes of immersion oils due to aging and evaporation are beneficial for tracking long-term live cell dynamics using microscopy imaging.

## Data Availability

The data presented in this study are available on request from the corresponding author.

## Supplementary Materials

The following supporting information can be downloaded at: https://www.mdpi.com/article/10.3390/mi13040576/s1, Video S1: The time-lapse movie of the expansion of bacteria bearing green or red fluorescence taken with Nikon F immersion oil. The scale bar corresponds to 10 μm. The focus slowly deviates and eventually causes single bacteria segmentation difficulty after around 15 h. Video S2: The time-lapse movie of the expansion of bacteria bearing green or red fluorescence taken with Millipore immersion oil. The scale bar indicates 10 μm. The focus locking is more stable throughout the experiment and allows long-term tracking and analysis. Video S3: The time-lapse movie of the expansion of *E. coli* expressing GFP for 80 h with stable focus taken with Millipore immersion oil. The scale bar indicates 10 μm.

## Abbreviations

The following abbreviations are used in this manuscript:

ANOVA	Analysis of variance
CFD	Computational fluid dynamics
FEM	Finite element method
FVM	Finite volume method
GFP	Green fluorescence protein
PDMS	Poly(dimethyl)siloxane
PMMA	Polymethylmethacrylate
